# Prognostic role of c‐Jun activation domain‐binding protein‐1 in cancer: A systematic review and meta‐analysis

**DOI:** 10.1111/jcmm.16334

**Published:** 2021-02-07

**Authors:** Deyao Shi, Shidai Mu, Binwu Hu, Shuo Zhang, Jianxiang Liu, Zhicai Zhang, Zengwu Shao

**Affiliations:** ^1^ Department of Orthopaedics Union Hospital Tongji Medical College Huazhong University of Science and Technology Wuhan China; ^2^ Department of Hematology Union Hospital Tongji Medical College Huazhong University of Science and Technology Wuhan China

**Keywords:** biomarker, cancer, Jab1, meta‐analysis, prognosis

## Abstract

c‐Jun activation domain‐binding protein‐1 (Jab1) is aberrantly overexpressed in multiple cancers and plays an oncogenic role in cancer progression. We examined the association between Jab1 expression and prognosis in patients with cancer by conducting a meta‐analysis. A comprehensive search strategy was performed using the PubMed, Web of Science, Ovid and EMBASE in July 2020. Eligible studies were enrolled according to definite criteria. Twenty‐seven studies involving 2609 patients were enrolled in this meta‐analysis. A significant association between high Jab1 expression and poor overall survival (pooled hazard ratio [HR] 2.344, 95% confidence interval [CI]: 2.037‐2.696) was observed. Subgroup analyses of the type of cancer, sample size, follow‐up period, Jab1 detection method and preoperative treatment did not alter the significance. On pooling data from Cox multivariate analyses, high Jab1 expression was found to be an independent prognostic indicator for overall survival. In addition, high Jab1 expression was found to be associated with advanced clinicopathological features such as clinical stage, lymphatic metastasis, histological grade and distant metastasis in cancers. Our meta‐analysis is the first to demonstrate that high Jab1 expression may be a promising indicator of poor prognosis and has an independent prognostic value for overall survival in patients with cancer.

## 1 INTRODUCTION

Cancer is one of the leading causes of death worldwide. In 2020, more than 1.8 million new cancer cases and more than 600 000 cancer deaths were estimated to occur in the United States.^1^ Many patients with advanced cancer experience illness and a poor prognosis. Therefore, assessment and appropriate treatment strategies are required at an early stage. At present, many researchers are investigating biomarkers that may aid in the detection or evaluation of cancer.

c‐Jun activation domain‐binding protein‐1 (Jab1) is the fifth subunit of the constitutive photomorphogenic‐9 signalosome (COPS5/CSN5), a highly conserved protein complex that regulates a wide variety of cellular and developmental processes, such as signal transduction, cell proliferation, the cell cycle, apoptosis, DNA damage responses and tumorigenesis.^2^, ^3^ Increasing evidence indicates that dysregulation of Jab1/COPS5 contributes to tumorigenesis by functionally interacting with several tumour‐related proteins, such as the cyclin‐dependent kinase inhibitor 1B (p27) and 1C (p57), p53, SMAD4/7 and programmed death‐ligand 1 (PD‐L1).^4^, ^5^, ^6^, ^7^, ^8^, ^9^, ^10^ Based on data from the Gene Expression Profiling Interactive Analysis database (http://gepia.cancer‐pku.cn/index.html) and other related articles, Jab1/COPS5 overexpression has been reported in various cancers, including hepatocellular carcinoma (HCC), breast cancer, non‐small cell lung cancer and nasopharyngeal carcinoma^11^, ^12^, ^13^, ^14^, ^15^, ^16^ In addition, an increasing number of studies have revealed that Jab1 overexpression is associated with poor prognosis and clinicopathological characteristics in a variety of cancers, such as HCC, breast cancer and colorectal cancer.^12^, ^14^, ^17^

Hence, we expect Jab1 to act as a potential prognostic biomarker in cancer. Although numerous studies have assessed the prognostic value of Jab1 in multiple cancers, there are controversial results regarding overall survival (OS) or some clinicopathological characteristics. Thus, we performed a systematic review and meta‐analysis to evaluate the prognostic value of Jab1 expression in multiple cancers.

## 2 METHODS

### 2.1 Search strategy

In the present study, up‐to‐date databases including PubMed, Web of Science, Ovid and EMBASE were used for the selection of publications. We performed a publication search using the following keywords: ‘c‐Jun activation domain‐binding protein‐1 or Jab1 or constitutive photomorphogenic‐9 signalosome or CSN5 or COP9 Signalosome Subunit 5 or COPS5’ and ‘cancer or carcinoma or sarcoma or tumour or neoplasia or malignancy’ and ‘prognosis or outcome or survival or mortality or “hazard ratio” or HR’. The latest literature search was conducted on 15 July 2020.

### 2.2 Study selection criteria

The inclusion criteria for this study were as the following: (a) studies were published in English and full articles were available; (b) studies in which the clinical information of patients and expression levels of Jab1 were examined; (c) studies involving the statistical results of the association between Jab1 expression and patient prognosis; and (d) studies including the hazard ratios (HRs) and their 95% confidence intervals (CIs) for OS, progression‐free survival (PFS), disease‐free survival (DFS), relapse‐free survival (RFS), and metastasis‐free survival (MFS), or HRs and their 95% CIs that could be calculated from the data provided in the publications. The exclusion criteria for this study were as follows: (a) studies were in the form of review, case report or meta‐analysis; (b) studies have no available data of HR and 95% CI or the HR and 95% CI could not be calculated; (c) studies were evaluated as low quality; and (d) if duplicated clinical data were used in different studies, the prior or incomplete study would be excluded. Study selection was performed by two authors independently, and the divergence was solved by discussion in the group.

### 2.3 Quality assessment

Study quality was measured using the Newcastle‐Ottawa quality assessment scale (NOS). This scale contains a maximum score of 9 points for the assessment of each included study with 4 points in the selection, quality, 2 points on the comparability and 3 points on the quality of outcome and follow‐up. A study with a NOS score lower than 6 was considered to be of inferior quality and was excluded from this meta‐analysis. Study quality assessment was performed by two authors independently, and the divergence was solved by discussion in the group.

### 2.4 Data extraction

For each study, necessary information was extracted from the corresponding publication, including the first author's name, year of publication, country or region where the participants were enrolled, participants’ ethnicity, sample size, follow‐up period, the detection method for Jab1, cut‐off value, tumour stage, pre‐operation treatment, prognosis assessment (OS, PFS, DFS, RFS or MFS), HRs with their 95% CIs and *P* value, statistical method of the study. Besides, for any study in which the HRs with their 95% CIs and *P* values could not be obtained directly from the publication, these important data were obtained through the following approaches: (a) clinical information and corresponding statistical data were acquired by contacting the corresponding author; and (b) HRs with 95% CIs were calculated according to a previously reported^18^ method based on the total number of events, *P* values and extraction of data from the Kaplan‐Meier curves in the original publication.

### 2.5 Statistical analysis

In this meta‐analysis, we used Stata 14.0 (STATA Corporation, College Station, TX, USA) to analyse the data. HRs with their 95% CIs were used to assess the association between Jab1 expression and patient prognosis. An HR > 1 indicated a poor prognosis in patients. For the pooled results, we applied the chi‐square‐based Q test and Higgins *I* ^2^ statistic to evaluate heterogeneity. A *P* value lower than 0.05 and *I* ^2^ value larger than 50% implied that there was significant heterogeneity. The fixed‐effects model was used when no significant heterogeneity was observed; otherwise, a random‐effect model was applied. Publication bias was evaluated using Egger's and Begg's tests. When significant publication bias was observed, ‘trim and fill’ analysis was used to detect and adjust for publication bias in the meta‐analysis results.^19^, ^20^ Besides, in the process of data extraction from Kaplan‐Meier curve, the Engauge Digitizer (Version 4.0, http://engauge‐digitizer.software.informer.com) was used.

## 3 RESULTS

### 3.1 Data selection and characteristics of studies

**FIGURE 1:**
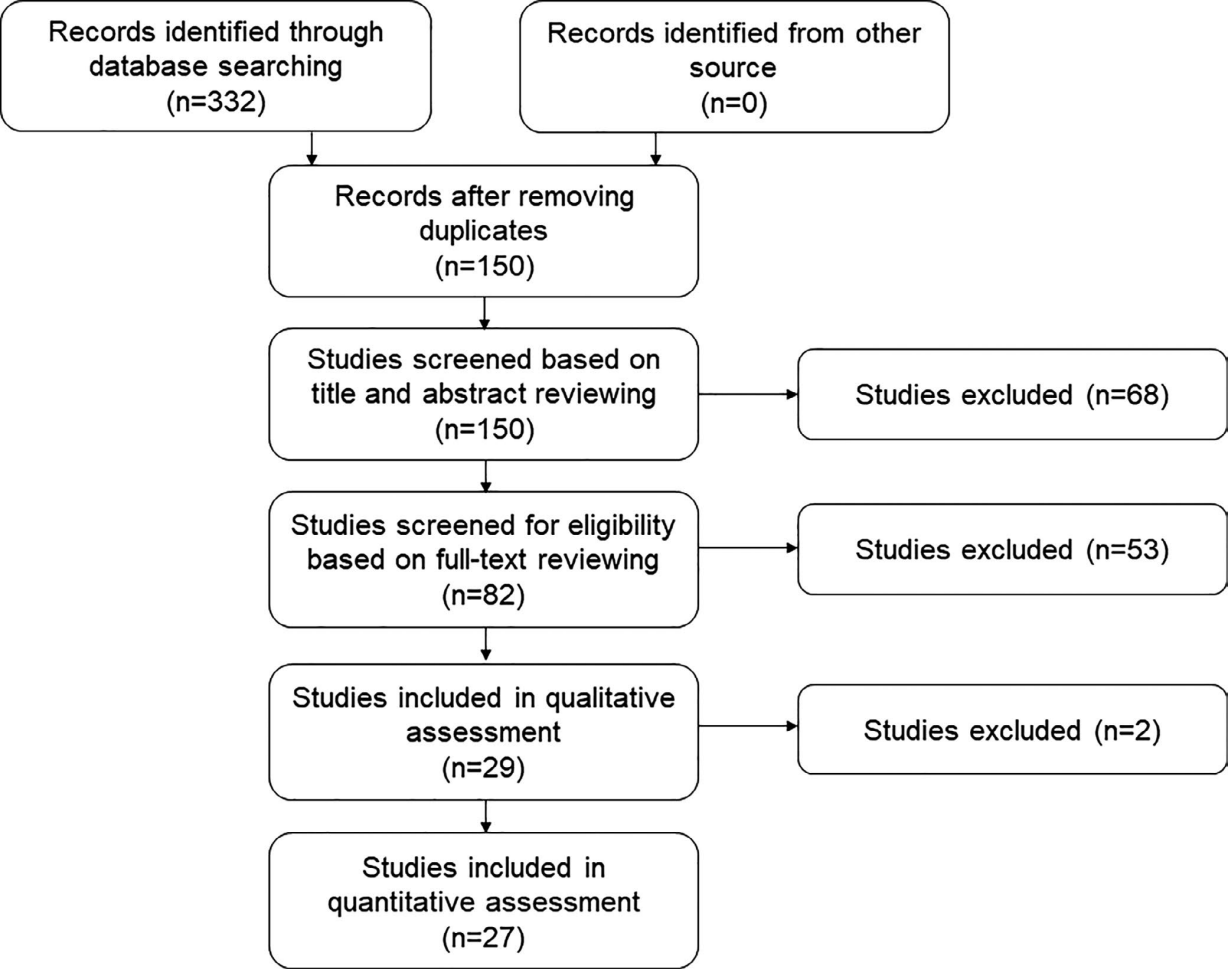
The flow diagram of the meta‐analysis

We used the designed search strategy to collect 498 studies. After excluding duplicates, 152 articles remained. Based on the inclusion and exclusion criteria described above, 27 studies were finally enrolled. The detailed process of study selection is shown in Figure 1.

In Table 1, the characteristics of each enrolled study are presented. In summary, this meta‐analysis evaluated 27 studies involving a total of 2609 patients. The mean sample size of the enrolled studies was 97 (range: 45‐206). The publication time of the studies ranged from 2001 to 2020. The patients involved in this meta‐analysis were from China, Japan, Norway and Russia. Seventeen types of malignancies were evaluated. Among them, OS, RFS, DFS and PFS were utilized to evaluate survival outcomes in 26, 1, 2 and 2 studies, respectively. All of the studies performed Kaplan‐Meier survival analysis, and 16 studies applied Cox proportional hazard regression for survival analysis. In addition to the survival data, 19 studies contained data on the correlation between Jab1 expression and clinicopathological characteristics such as clinical stage, tumour histological grade and lymph node metastasis.

**TABLE 1:** Characteristics of studies enrolled in the meta‐analysis

Study	Year	Region	Cancer type	Sample size	Follow‐up (mo)	Detection method	Cut‐off value (Jab1 expression: High vs Low)	Tumour stage	Pre‐operation treatment	Survival outcome	Survival analysis	Method	NOS score
Sui et al^33^	2001	Japan	Ovarian cancer	47	156	IHC	Stained 10%	I‐IV	No	OS	Kaplan‐Meier plot, Cox proportional hazard regression	1	8
Shintani et al^34^	2003	Japan	Oral squamous cell carcinoma	75	110	IHC	Stained 10%	I‐IV	Chemotherapy (stage III‐IV)	OS	Kaplan‐Meier plot	2	6
Dong et al^35^	2005	China	Laryngeal Squamous Cell Carcinoma	102	60	IHC	Stained 50%	I‐IV	No	OS, DFS	Kaplan‐Meier plot, Cox proportional hazard regression	1	8
Osoegawa et al^36^	2006	Japan	Non‐small cell lung cancer	138	Over 60	IHC	Stained 50%	I‐IV	NA	OS	Kaplan‐Meier plot, Cox proportional hazard regression	1	7
Harada et al^37^	2006	Japan	Oral squamous cell carcinoma	102	100	IHC	Stained 50%	II‐IV	Chemotherapy and radiotherapy	OS	Kaplan‐Meier plot, Cox proportional hazard regression	1	7
Wang et al^38^	2008	China	Non‐Hodgkin lymphoma	51	60	IHC	Stained 10%	I‐IV	NA	OS	Kaplan‐Meier plot	2	6
Hashimoto et al^39^	2009	Japan	Cholangiocarcinoma	74	Over 60	IHC	Stained 30%	NA	No	OS	Kaplan‐Meier plot, Cox proportional hazard regression	2	7
Chen et al^40^	2010	China	Hepatocellular carcinoma	76	Over 60	IHC	Stained 69%	NA	NA	OS	Kaplan‐Meier plot	2	7
Gao et al^41^	2012	China	Oral squamous cell carcinoma	206	60	IHC	Stained 10%	I‐IV	No	OS	Kaplan‐Meier plot	2	6
He et al^42^	2012	China	Glioma	192	120	IHC	Stained 10%	II‐IV	No	OS	Kaplan‐Meier plot, Cox proportional hazard regression	1	7
Sorbye et al^43^	2012	Norway and Russia	Soft tissue sarcoma	178	392	IHC	Specific staining scoring (score = 2)	NA	NA	OS	Kaplan‐Meier plot	2	6
Pan et al^16^	2012	China	Nasopharyngeal carcinoma	45	60	IHC	Specific staining scoring (score = 2.5)	I‐IV	No	OS	Kaplan‐Meier plot	2	6
Wang et al^14^	2014	China	Breast cancer	95	120	IHC	Stained 27%	NA	NA	OS	Kaplan‐Meier plot, Cox proportional hazard regression	1	7
Wang et al^12^	2014	China	Hepatocellular carcinoma	76	60	IHC	Specific staining grading (grade = 4)	NA	No	OS	Kaplan‐Meier plot, Cox proportional hazard regression	1	7
Guo et al^7^	2016	China	Hepatocellular carcinoma	67	Over 100	IHC	Median	I‐IV	No	OS	Kaplan‐Meier plot	2	7
Ma et al^44^	2016	China	Non‐Hodgkin lymphoma	50	Over 80	IHC	Specific staining scoring (score = 4)	NA	NA	OS	Kaplan‐Meier plot, Cox proportional hazard regression	1	7
Kugimiya et al^17^	2017	Japan	Colorectal cancer	57	38.3	IHC	ROC Curve	II‐III	Chemotherapy	RFS	Kaplan‐Meier plot, Cox proportional hazard regression	1	8
Zhou et al^45^	2017	China	Colorectal cancer	113	Over 120	IHC	Stained (++)	I‐IV	NA	OS	Kaplan‐Meier plot, Cox proportional hazard regression	1	7
Liu et al^29^	2017	China	Hepatocellular carcinoma	102	Over 80	IHC	NA	I‐III	NA	OS	Kaplan‐Meier plot	2	6
Zhou et al^46^	2017	China	Acute monocytic leukaemia	60	60	Western Blotting	Median	NA	Chemotherapy	OS	Kaplan‐Meier plot, Cox proportional hazard regression	1	7
Zhang et al^28^	2017	China	Renal cell carcinoma	80	Over 150	IHC	NA	I‐IV	NA	OS	Kaplan‐Meier plot	2	6
Xiao et al^47^	2018	China	Non‐small cell lung cancer	59	Over 80	IHC	Specific staining scoring (score = 6)	I‐III	No	OS, PFS	Kaplan‐Meier plot, Cox proportional hazard regression	1	8
Li et al^15^	2018	China	Non‐small cell lung cancer	102	Over 90	Western Blotting	NA	I‐IV	NA	OS, DFS	Kaplan‐Meier plot	2	6
Wu et al^48^	2019	China	Breast cancer	140	Over 120	IHC	Median	NA	NA	OS	Kaplan‐Meier plot	2	6
Wan et al^49^	2019	China	Osteosarcoma	108	60	IHC	NA	I‐IV	NA	OS, PFS	Kaplan‐Meier plot, Cox proportional hazard regression	1	7
Shen et al^50^	2020	China	Oesophageal squamous cell carcinoma	124	64	IHC	NA	I‐IV	No	OS	Kaplan‐Meier plot, Cox proportional hazard regression	1	7
Wang et al^51^	2020	China	Gastric cancer	90	Over 60	IHC	Specific immunoreactive scoring (score = 8)	I‐IV	NA	OS	Kaplan‐Meier plot, Cox proportional hazard regression	1	8

Note

Method: *1 denoted as obtaining HRs and 95% CIs directly from publications; 2 denoted as obtaining HRs and 95% CIs based on the provided data and extraction of Kaplan‐Meier curves from publications.

Abbreviations: DFS, disease‐free survival; IHC, Immunohistochemistry; NOS, Newcastle‐Ottawa Scale; OS, overall survival; PFS, progression‐free survival; RFS, recurrence‐free survival; WB, Western Blotting.

### 3.2 Association of Jab1 expression with prognosis

**FIGURE 2:**
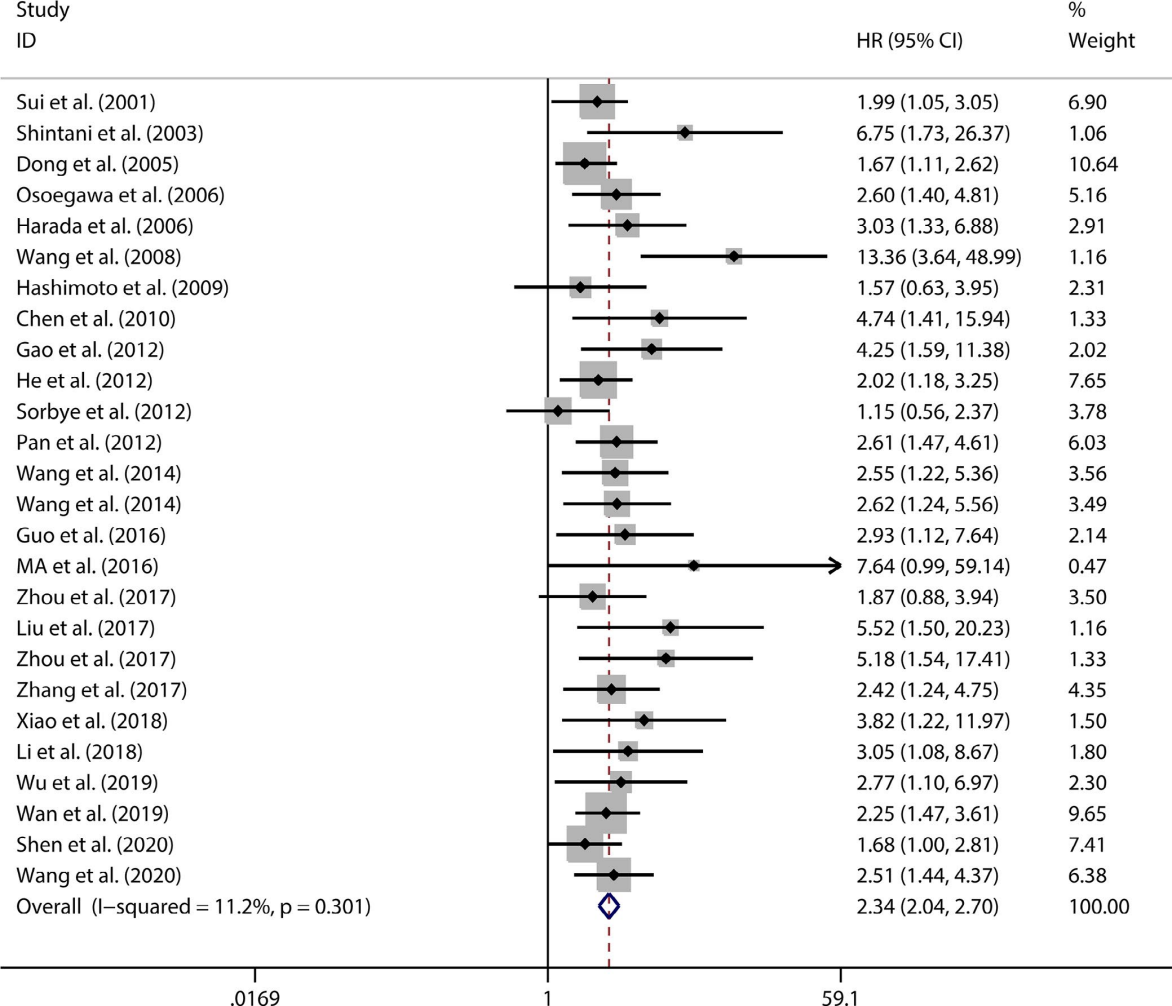
Forest plot of overall survival according to Jab1 expression in cancers

**FIGURE 3:**
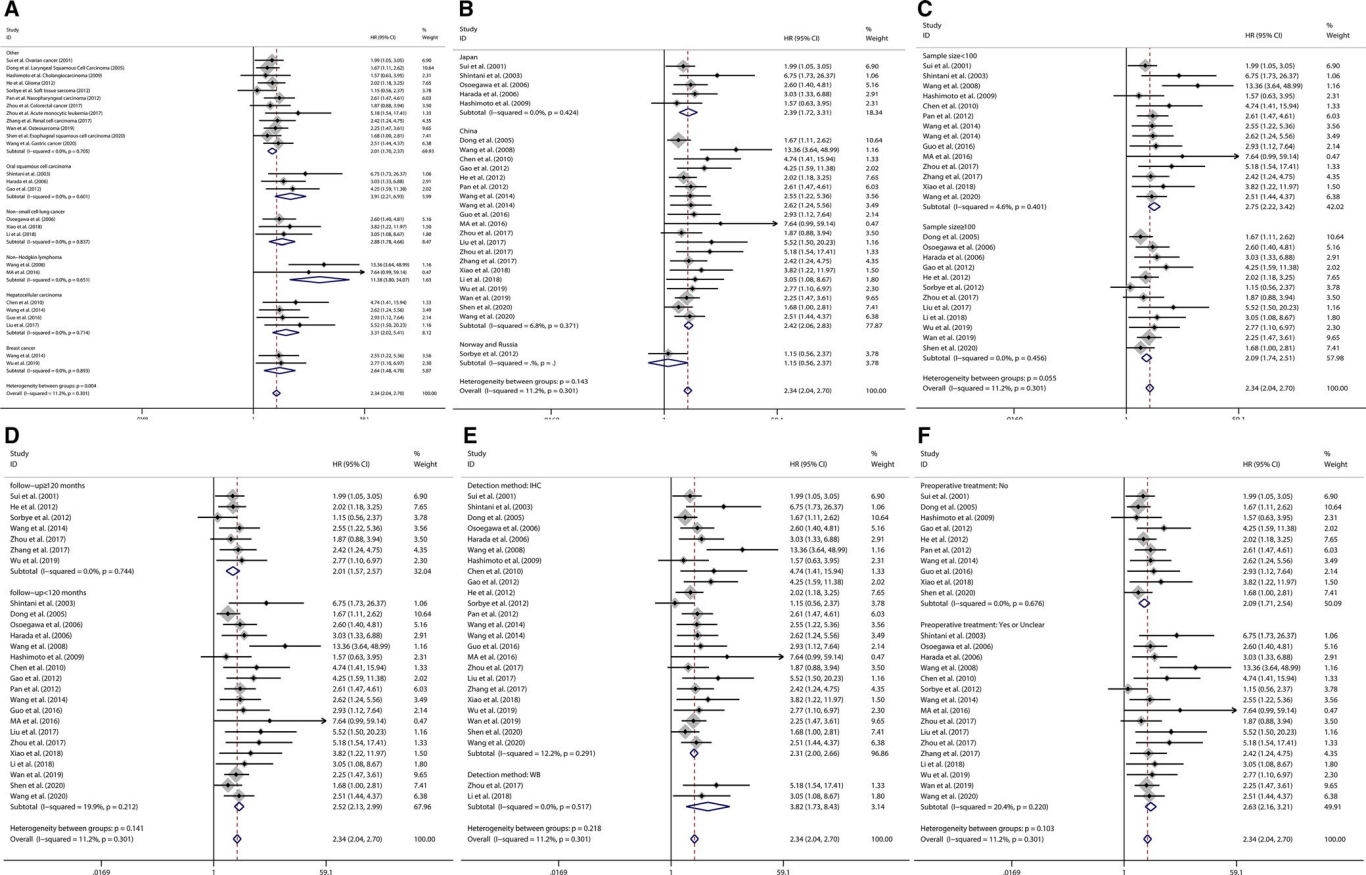
Forest plot of subgroup analysis for overall survival according to Jab1 expression. (A) type of cancer. (B) region of enrolled patients (C) sample size of study (D) follow‐up period. (E) Jab1 detection method (F) pre‐operation treatment

As shown in Figure 2, 26 studies demonstrated an association between OS and Jab1 expression involving 17 types of cancers in 2552 patients. The pooled HR and corresponding 95% CI were 2.344 (2.037‐2.696) (Jab1 expression: high vs low), indicating a significant association between Jab1 overexpression and poor OS in patients with cancer. A fixed‐effects model was used because of the low heterogeneity (χ^2^ = 28.15, *df* = 25, *P* = 0.301; *I* ^2^ = 11.2%). Subsequent subgroup analysis was carried out according to the type of cancer, region of the enrolled patients, sample size, follow‐up period, Jab1 detection method and preoperative treatment (Table 2 and Figure 3). As shown in the pooled HR with 95% CI and heterogeneity in each subgroup analysis, each of these factors did not alter the association between Jab1 overexpression and OS in patients with cancer, except in those in Norway and Russia.

**TABLE 2:** Subgroup analysis of association between overall survival and high Jab1 expression

Subgroup analysis	No. of studies	No. of patients	HR (95% CI)	Heterogeneity (*P*‐value, *I* ^2^)	Model
Type of cancer					
Breast cancer	2	235	2.636 (1.478‐4.700)	0.893, 0.0%	Fixed
Hepatocellular carcinoma	4	321	3.308 (2.023‐5.409)	0.714, 0.0%	Fixed
Non‐Hodgkin lymphoma	2	101	11.376 (3.798‐34.072)	0.651, 0.0%	Fixed
Non‐small cell lung cancer	3	299	2.879 (1.779‐4.6590	0.837, 0.0%	Fixed
Oral squamous cell carcinoma	3	383	3.911 (2.207‐6.932)	0.601, 0.0%	Fixed
Other	12	1213	2.006 (1.697‐2.372)	0.705, 0.0%	Fixed
Region					
China	20	1938	2.415 (2.061‐2.831)	0.371, 6.8%	Fixed
Japan	5	436	2.388 (1.722‐3.312)	0.424, 0.0%	Fixed
Norway and Russia	1	178	1.152 (0.560‐2.367)	—	—
Sample size					
Sample size < 100	14	945	2.753 (2.218‐3.417)	0.401, 4.6%	Fixed
Sample size ≥ 100	12	1607	2.086 (1.735‐2.507)	0.456, 0.0%	Fixed
Follow‐up period					
Follow‐up < 120 months	19	1707	2.519 (2.126‐2.986)	0.212, 19.9%	Fixed
Follow‐up ≥ 120 months	7	845	2.010 (1.570‐2.575)	0.744, 0.0%	Fixed
Detection method of Jab1					
IHC	24	1290	2.307 (2.001‐2.660)	0.291, 12.2%	Fixed
WB	2	162	3.823 (1.734‐8.432)	0.517, 0.0%	Fixed
Pre‐operation treatment					
No	10	992	2.086 (1.711‐2.543)	0.676, 0.0%	Fixed
Yes or Unclear	16	1560	2.634 (2.160‐3.212)	0.220, 20.4%	Fixed

Moreover, RFS, DFS and PFS were used as survival outcomes in a total of five studies with 428 patients (Table 3). Although a pooled analysis was not performed due to the limited number of related studies, it is noteworthy that Jab1 overexpression was not correlated with DFS of patients with laryngeal squamous cell carcinoma in Dong et al’s study; however, the other four studies indicated Jab1 overexpression as a prognostic factor for RFS, DFS and PFS in cancers.

**TABLE 3:** High Jab1 expression and other survival outcome in cancers

Study year	Cancer type	Region	Survival analysis	HR (95% CI)
RFS				
Kugimiya et al 2017	Colorectal cancer	Japan	Kaplan‐Meier plot and Cox proportional hazard regression	2.80 (1.070‐8.780)
DFS				
Dong et al 2015	Laryngeal Squamous Cell Carcinoma	China	Kaplan‐Meier plot and Cox proportional hazard regression	1.310 (0.930‐1.880)
Li et al 2018	Non‐small cell lung cancer	China	Kaplan‐Meier plot	3.843 (1.421‐10.392)
PFS				
Wan et al 2019	Osteosarcoma	China	Kaplan‐Meier plot	2.444 (1.001‐5.971)
Xiao et al 2018	Non‐small cell lung cancer	China	Kaplan‐Meier plot	4.895 (1.514‐15.830)

### 3.3 Independent prognostic value of Jab1 expression in OS

**FIGURE 4:**
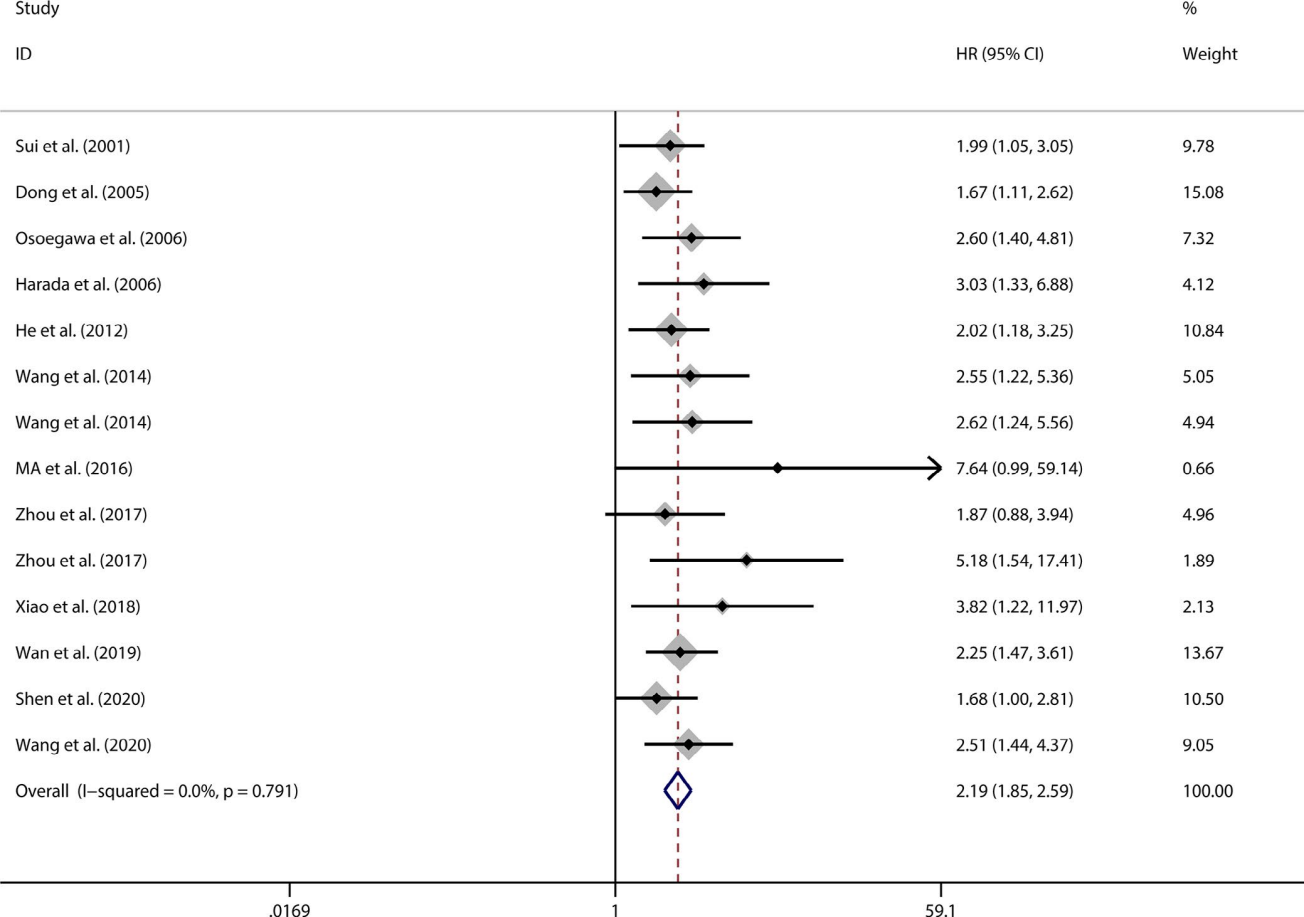
Forest plot for meta‐analysis of the independent role of Jab1 expression in overall survival

Among the 27 included studies, a total of 14 studies used a Cox proportional hazard model for multivariate analysis, which enrolled 13 types of cancer with 1356 patients. As shown in Figure 4, we observed a significant association between Jab1 overexpression and poor OS in patients with multiple cancers (pooled HR 2.190, 95%CI: 1.853‐2.587). A fixed‐effects model was used because low heterogeneity was detected among studies (χ^2^ = 8.76, *df* = 13, *P* = 0.791; *I* ^2^ = 0.0%). This result indicated that Jab1 overexpression may act as an independent prognostic factor for OS in human cancers.

### 3.4 Association between Jab1 expression and clinicopathological characteristics of cancers

**FIGURE 5:**
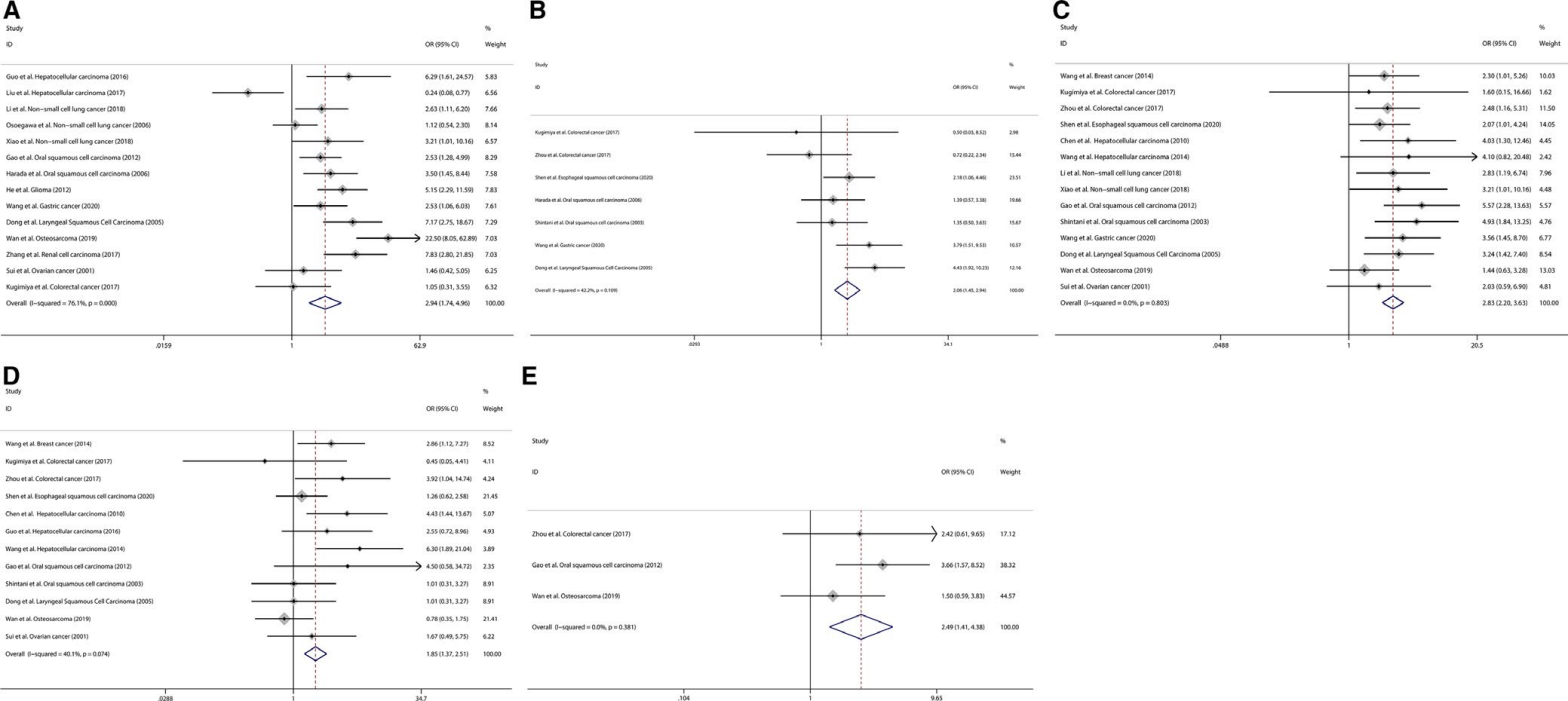
Forest plot of clinicopathological characteristics according to Jab1 expression in cancers. A, clinical staging. B, size or invasion extent of the primary tumour. C, lymphatic metastasis. D, histological grade. E, distant metastasis

In the present meta‐analysis, clinicopathological characteristics including clinical staging, size or invasion extent of the primary tumour, lymphatic metastasis, histological grade and distant metastasis were analysed for the correlation with Jab1 expression (Figure 5 and Table 4). Fourteen studies involving 10 types of cancer with 1452 patients showed a significant association between high Jab1 expression and advanced clinical stages (pooled OR 2.939, 95%CI: 1.742‐4.960). Seven studies involving five types of cancer with 663 patients presented a significant association between high Jab1 expression and enlarged size or invasion extent of the primary tumours (pooled OR 2.062, 95%CI: 1.448‐2.937). A significant association between Jab1 overexpression and lymphatic metastasis was observed in 14 studies involving 10 types of cancer in 1330 patients (pooled OR 2.829, 95%CI: 2.202‐3.634). Twelve studies involving eight types of cancer with 1146 patients showed a significant association between high Jab1 expression and poor histological grade of tumours (pooled OR 1.850, 95%CI: 1.366‐2.506). And a significant association between Jab1 overexpression and distant metastasis of tumours was observed in three studies involving three types of cancer with 427 patients (pooled OR 2.487, 95%CI: 1.413‐4.377). In summary, these results suggest that high Jab1expression is significantly associated with advanced clinicopathological features of human cancers.

**TABLE 4:** Meta‐analysis of the association between Jab1 expression and clinicopathological characteristics of cancers

Clinicopathological characteristics	No. of studies	No. of patients	No. of cancer types	OR (95% CI)	Heterogeneity (*P*‐value, *I* ^2^)	Model
Clinical staging: III/IV vs I/II	14	1452	10	2.939 (1.742‐4.960)	<0.001, 76.1%	Random
Size or invasion extent of the primary tumours: T3/4 vs T1/2	7	663	5	2.062 (1.448‐2.937)	0.109, 42.2%	Fixed
Lymphatic metastasis: Nx‐N3 vs N0	14	1330	10	2.829 (2.202‐3.634)	0.803, 0.0%	Fixed
Histological grade: Poor vs Well/Moderate	12	1146	8	1.850 (1.366‐2.506)	0.074, 40.1%	Fixed
Distant metastasis: Mx/M1 vs M0	3	427	3	2.487 (1.413‐4.377)	0.381, 0.0%	Fixed

### 3.5 Sensitivity analysis and publication bias

**FIGURE 6:**
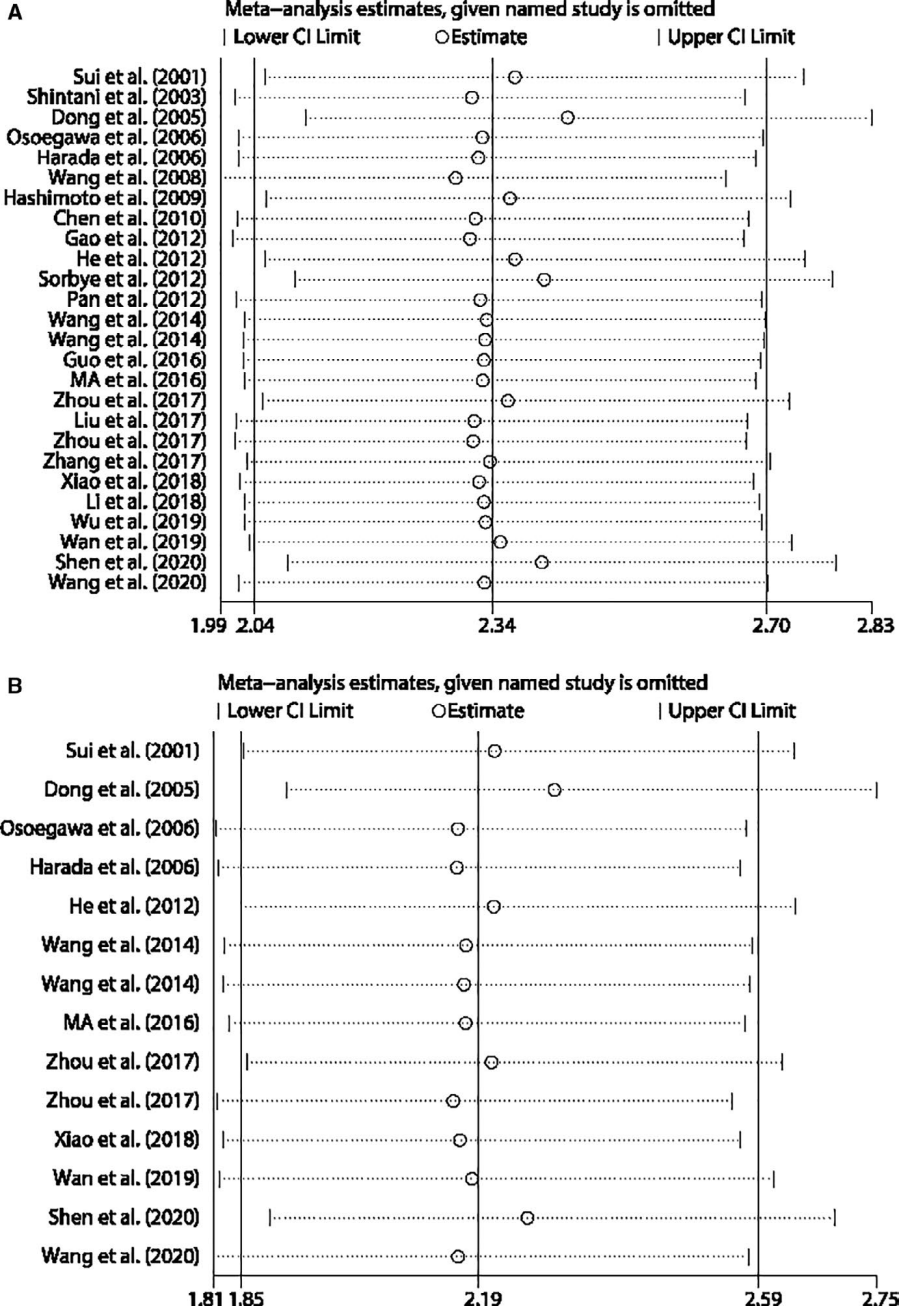
A, Sensitivity analysis (influence analysis) for the analysis of overall survival according to Jab1 expression. B, Sensitivity analysis (influence analysis) for the independent role of Jab1 expression in overall survival

Sensitivity analysis was performed to further assess the stability of the obtained results, although no significant heterogeneity was observed in the analysis of the association between Jab1 expression and OS. As shown in Figure 6A, exclusion of any individual study did not alter the significance of the association between Jab1 expression and OS. Similarly, sensitivity analysis was applied to studies that performed Cox multivariate analysis. Excluding any individual study did not influence the significance of the independent prognostic value of Jab1 expression in OS (Figure 6B).

**FIGURE 7:**
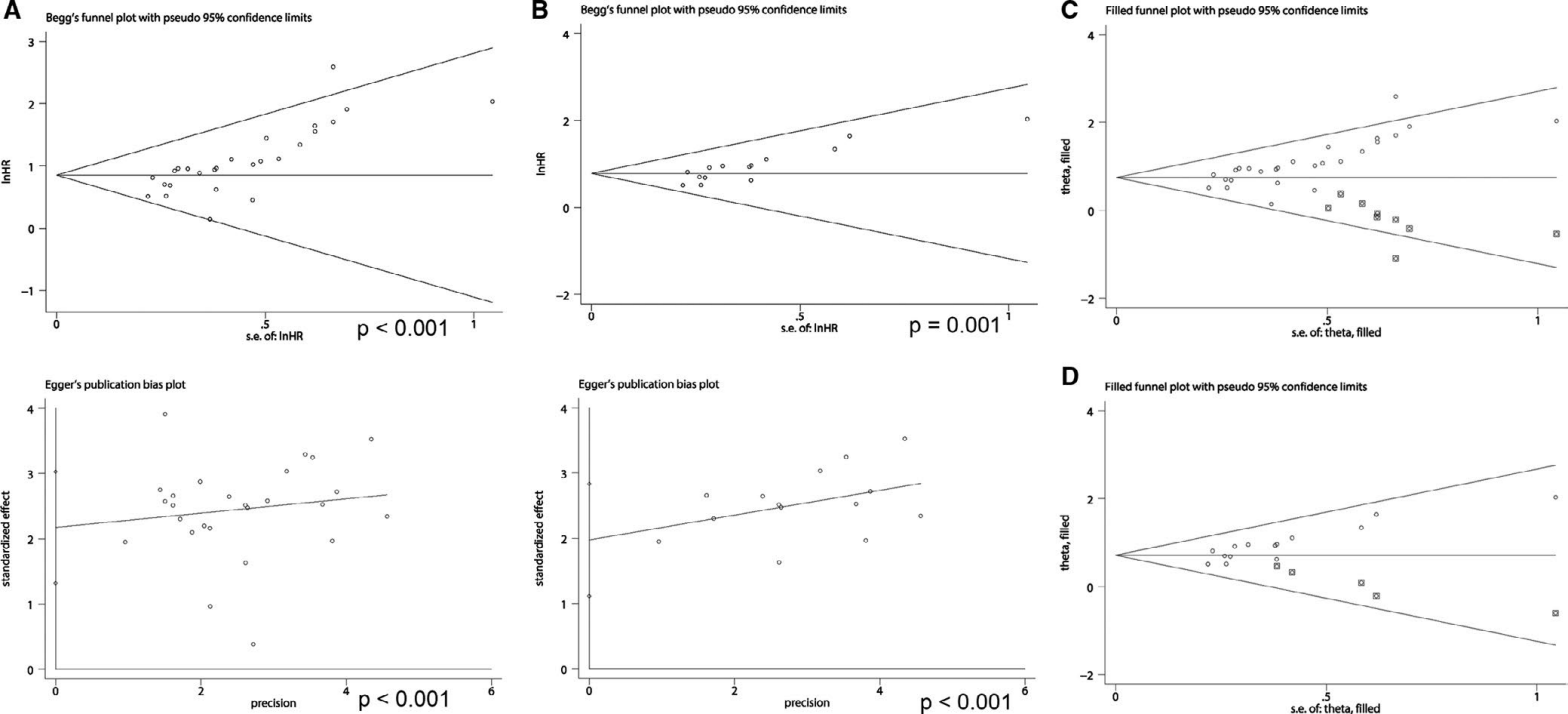
A, Publication bias detection for the analysis of overall survival according to Jab1 expression. B, Publication bias detection for the independent role of Jab1 expression in overall survival. C, Filled funnel plot of meta‐analysis using the ‘trim‐and‐fill’ method for the analysis of overall survival according to Jab1 expression. D, Filled funnel plot of meta‐analysis using the ‘trim‐and‐fill’ method for the independent role of Jab1 expression in overall survival

For publication bias, Begg's funnel plot and Egger's linear regression tests were performed and both showed significant evidence of publication bias among our pooled results. The analysis of the association between Jab1 expression and OS revealed *P* < 0.001 in Begg's test and *P* < 0.001 in Egger's test, as shown in Figure 7A. The analysis of the independent prognostic value of Jab1 expression in OS revealed *P* = 0.001 in Begg's test and *P* < 0.001 in Egger's test (Figure 7B). Thus, for the analysis of the association between Jab1 expression and OS, we carried out nonparametric ‘trim‐and‐fill’ analysis as shown in Figure 7C, which detected nine trimmed studies, and the estimated pooled HR and 95% CI were 2.114 (1.852‐2.413) after adjustment. For the analysis of the independent prognostic value of Jab1 expression in OS, we found that five studies were trimmed and the estimated pooled HR with its 95% CI was 2.046 (1.750‐2.392) after adjustment (Figure 7D).

Sensitivity analysis and assessment of publication bias were also conducted to analyse the association between Jab1 expression and clinicopathological characteristics of cancers. For the results of sensitivity analysis, exclusion of any individual study did not alter the significance of the association between Jab1 expression and each analysed clinicopathological characteristic, except for distant metastasis (Figure S1). For the results of publication bias assessment, neither Begg's funnel plot nor Egger's linear regression test found significant publication bias in any analysis of each clinicopathological characteristic (Figure S2).

## 4 DISCUSSION

As one of the major public health problems worldwide, cancer causes a large number of deaths and creates a significant economic burden every year. The exploration of accurate and effective indicators for diagnosis and prognosis has become a major objective in oncology research.

Over the past few years, an increasing number of studies have demonstrated that Jab1 is aberrantly overexpressed in multiple cancers and plays an important role in regulating tumorigenesis and tumour progression. Jab1 lies at the intersection of several important signal transduction pathways. Various upstream signalling pathways have been reported to regulate the expression of Jab1, such as IL6‐Stat3, HER‐2, EGFR, TGF‐β, Wnt/β‐catenin, NF‐κB, MIF‐PI3K‐AKT and BCR‐ABL signallings.^5^, ^21^, ^22^, ^23^, ^24^, ^25^, ^26^, ^27^ In addition, Jab1 has multiple functional downstream targets, including p27, PD‐L1, NcoR, MDM2 and Bcl2. Jab1 exerts notable effects on diverse cellular functions such as cell cycle progression, apoptosis, DNA damage repair, angiogenesis, senescence and reactive oxygen species regulation in the development and progression of tumours.^4^, ^5^, ^6^, ^7^, ^8^, ^9^, ^10^

In addition, accumulating studies have demonstrated a correlation between Jab1 overexpression and poor prognosis in patients with cancer. In Pan et al’s study,^16^ Jab1 was found to interact directly with p27 and mediate p27 degradation in a proteasome‐dependent manner, and high Jab1 expression was found to be associated with poor prognosis in patients with nasopharyngeal carcinoma. Zhang et al^28^ found that Jab1 promotes epithelial‐mesenchymal transition by inhibiting ZEB1 degradation, and Jab1 overexpression is correlated with poor OS in patients with renal cell carcinoma. Liu et al’s study^29^ showed that Jab1 contributes to chemoresistance in breast cancer by regulating Rad51, and patients with Jab1 overexpression exhibited a poor prognosis. Kugimiya et al’s study^17^ on colorectal cancer verified Jab1 as a prognostic biomarker and the JAB1‐STAT3 over‐activation loop as playing a role in the recurrence of colorectal cancer following adjuvant chemotherapy.

Increasing evidence suggests that Jab1/COPS5 is a promising therapeutic target, given its multiple prominent functions during tumorigenesis and the significant correlation between Jab1 overexpression and poor prognosis. Several compounds, such as curcumin and its analogs, troglitazone, doxycycline and thiolutin, have been shown to inactivate Jab1/COPS5 and/or reduce its expression, thus attenuating tumour growth.^30^ Moreover, a highly specific small‐molecule inhibitor of Jab1/CSN5 (CSN5i‐3) was recently reported to have a large anti‐tumour therapeutic window according to recent studies that involved in vitro and in vivo tests.^31^, ^32^

The present meta‐analysis is the first study to summarize the effect of Jab1 as a prognostic indicator in various cancers, enrolling 27 independent studies on 17 types of human malignancies with 2609 patients. Among these studies, Jab1 was overexpressed in tumour tissue compared to that in adjacent normal tissue. By merging the data of survival analysis, we found that high Jab1 expression is associated with poor prognosis in terms of OS in patients with cancer with no significant heterogeneity. Subsequently, subgroup analysis of the type of cancer, region of the enrolled patients, sample size, follow‐up period, Jab1 detection method and preoperative treatment showed that a significant association between Jab1 overexpression and short OS would not be altered by these factors, except Norway and Russia. It should be noted that the individual study by Sorbye et al demonstrated that Jab1 expression is not correlated with OS in patients with soft tissue sarcoma. In addition, we combined the HRs of 14 studies using the Cox proportional hazard model for multivariate analysis of the survival data and found that Jab1 overexpression acts as an independent prognostic indicator for OS in human cancers (pooled HR 2.190, 95%CI: 1.853‐2.587) with low heterogeneity detected. The stability of these results was verified by the sensitivity analysis. No alteration of the pooled results was observed by excluding any individual study. However, significant publication bias was found, thus nonparametric ‘trim‐and‐fill’ analysis was used for detection and estimation. The estimated pooled HRs and 95% CIs showed no significant alteration in these results after the trim‐and‐fill adjustment. Therefore, our results provide robust evidence that high Jab1 expression is significantly associated with poor OS and may act as an independent prognostic indicator of OS in patients with cancer.

In addition, we collected other survival data, such as RFS, DFS and PFS, from five studies and found that high Jab1 expression is associated with poor RFS, DFS and PFS in patients with colorectal cancer, non‐small cell lung cancer and osteosarcoma respectively, although Jab1 expression was reported to not be correlated with DFS in patients with laryngeal squamous cell carcinoma according to Dong et al’s study.

Through a systematic review, we found 14 studies involving 10 types of cancer with 1452 patients revealing data regarding the correlation between Jab1 expression and clinicopathological characteristics. Our pooled results suggest that high Jab1 expression is associated with advanced clinical staging, enlarged size or invasion extent of the primary tumour, positive lymphatic metastasis, poor histological grade and positive distant metastasis. The robustness of our results was based on the absence of significant alterations in the sensitive analysis and lack of significant publication bias. Thus, Jab1 overexpression may act as an indicator of prognosis and advanced clinicopathological characteristics in cancers.

It is noteworthy that there were several limitations to our study. First, only articles written in English were included, most of which were from China and Japan. Data corresponding to non‐Asian patients are limited. Second, overestimation of the prognostic value of Jab1 was inevitable because a small number of publications reported negative results. Third, the HRs and 95% CIs in several studies were only obtained by extraction and calculation. Fourth, the cut‐off values of high and low expression of Jab1 were diverse among studies. Hence, more well‐designed, large‐scale studies are warranted to confirm our results.

In summary, this meta‐analysis is the first to demonstrate that Jab1 is a promising indicator of prognosis in human cancers. Our study found that high Jab1 expression is associated with poor OS and advanced clinicopathological characteristics and possesses an independent prognostic value for OS in patients with cancer. In the future, more further studies of Jab1 in cancers should be conducted to investigate the underlying biological mechanisms, novel therapeutic targets and clinical translational applications.

## CONFLICT OF INTEREST

The authors declare that there is no conflict of interest.

## AUTHOR CONTRIBUTIONS

**Deyao Shi:** Data curation (lead); Formal analysis (lead); Investigation (lead); Methodology (equal); Software (lead); Validation (equal); Writing‐original draft (lead). **Shidai Mu:** Data curation (supporting); Formal analysis (supporting); Investigation (supporting). **Binwu Hu:** Data curation (supporting); Investigation (supporting). **Shuo Zhang:** Formal analysis (supporting); Investigation (supporting). **Jianxiang Liu:** Formal analysis (supporting); Investigation (supporting); Methodology (supporting). **Zhicai Zhang:** Conceptualization (equal); Validation (equal); Writing‐review & editing (equal). **Zengwu Shao:** Conceptualization (equal); Methodology (equal); Validation (lead); Writing‐review & editing (lead).

## Supporting information

Supporting Information

## Data Availability

The datasets used and/or analysed during the current study are available from the corresponding author on reasonable request.
